# An international collaborative family-based whole genome quantitative trait linkage scan for myopic refractive error

**Published:** 2012-03-26

**Authors:** Diana Abbott, Yi-Ju Li, Jeremy A. Guggenheim, Ravikanth Metlapally, Francois Malecaze, Patrick Calvas, Thomas Rosenberg, Sandrine Paget, Tetyana Zayats, David A. Mackey, Sheng Feng, Terri L. Young

**Affiliations:** 1 Center for Human Genetics, Duke University Medical Center, Durham, NC; 2 Department of Biostatistics and Bioinformatics, Duke University Medical Center, Durham, NC; 3 School of Optometry and Vision Sciences, Cardiff University, Cardiff, Wales, United Kingdom; 4 Department of Ophthalmology, Duke University Eye Center, Durham, NC; 5 Toulouse University Hospital, Toulouse, France; 6 Kennedy Centre, Gordon Norrie Centre and National Eye Clinic, Glostrup, Denmark; 7 Centre for Eye Research Australia, Department of Ophthalmology, University of Melbourne, Melbourne, Australia

## Abstract

**Purpose:**

To investigate quantitative trait loci linked to refractive error, we performed a genome-wide quantitative trait linkage analysis using single nucleotide polymorphism markers and family data from five international sites.

**Methods:**

Genomic DNA samples from 254 families were genotyped by the Center for Inherited Disease Research using the Illumina Linkage Panel IVb. Quantitative trait linkage analysis was performed on 225 Caucasian families and 4,656 markers after accounting for linkage disequilibrium and quality control exclusions. Two refractive quantitative phenotypes, sphere (SPH) and spherical equivalent (SE), were analyzed. The SOLAR program was used to estimate identity by descent probabilities and to conduct two-point and multipoint quantitative trait linkage analyses.

**Results:**

We found 29 markers and 11 linkage regions reaching peak two-point and multipoint logarithms of the odds (LODs)>1.5. Four linkage regions revealed at least one LOD score greater than 2: chromosome 6q13–6q16.1 (LOD=1.96 for SPH, 2.18 for SE), chromosome 5q35.1–35.2 (LOD=2.05 for SPH, 1.80 for SE), chromosome 7q11.23–7q21.2 (LOD=1.19 for SPH, 2.03 for SE), and chromosome 3q29 (LOD=1.07 for SPH, 2.05 for SE). Among these, the chromosome 6 and chromosome 5 regions showed the most consistent results between SPH and SEM. Four linkage regions with multipoint scores above 1.5 are near or within the known myopia (MYP) loci of MYP3, MYP12, MYP14, and MYP16. Overall, we observed consistent linkage signals across the SPH and SEM phenotypes, although scores were generally higher for the SEM phenotype.

**Conclusions:**

Our quantitative trait linkage analyses of a large myopia family cohort provided additional evidence for several known MYP loci, and identified two additional potential loci at chromosome 6q13–16.1 and chromosome 5q35.1–35.2 for myopia. These results will benefit the efforts toward determining genes for myopic refractive error.

## Introduction

Myopia, or nearsightedness, is the most common human eye disorder. Its diagnosis is based on refractive error biometrics, either negative sphere (SPH) or spherical equivalent (SE) (SE=SPH^+^1/2 cylinder) and is measured in diopters (D). Worldwide, individuals do not share the same myopic development risk, as the prevalence of myopia varies in different countries. Studies, primarily in adults but in some schoolchildren cohorts, have reported approximate prevalence rates of 17% in Australia, 26%–35% in the United States, and 27% in Western Europe [1–4]. Higher prevalence rates of 71%–96% have been reported in Asian countries such as Japan, Taiwan, Hong Kong, and Singapore [5–7]. Having myopia can significantly impact one’s daily life. High-grade levels of myopic refractive error (e.g., SEM or SPH less than −5.00 D) have associated ocular comorbidities of increased risk of premature cataracts, glaucoma, retinal detachment, and macular chorioretinal degeneration [1,8–13]. Myopia clearly is a significant global public health problem.

The genetic basis of myopia is supported by familial aggregation, segregation, and twin studies. High heritability estimates have been reported for SEM (0.5–0.96) [14–18] and axial length (AL) (0.40–0.94) [14–18]. The relative risk of myopia in siblings of a person with myopia has been estimated to be 5–20 for high myopia (SEM ≤ −6.00 D), and 1.5–3 for lower degrees of myopia (SE: −1.00 to −3.00 D) [19,20]. To date, more than 18 myopia (MYP) genetic chromosomal loci (MYP1–MYP18 and other implicated chromosomal regions) have been reported by genome-wide linkage studies in families. Most regions (11 MYP loci) were mapped for high-grade myopia in limited pedigree linkage studies. Although quantitative refractive data are generally available on ascertained samples, very few studies have used these continuous traits to map quantitative trait loci (QTLs) for myopia.

Of the 18 MYP chromosomal loci identified, five (MYP7 [11p13], MYP8 [3q26], MYP9 [4q12], MYP10 [8p23], and MYP14 [1p34–36]) [21,22] were initially identified through quantitative trait linkage analysis using refractive error measurements (SPH and SE) as the traits. For example, the MYP7–MYP10 loci were identified using 221 dizygotic (DZ) twins from a classical twin study designed to investigate the heritability of refractive error. The strongest linkage signal was at chromosome 11p13, which contained the biologically relevant candidate gene paired box gene 6 (*PAX6*). In contrast to the twin study design, the MYP14 locus was a QTL found after 49 multigenerational Ashkenazi Jewish families ascertained for common myopia using SEM as the trait were analyzed [22]. No evidence of linkage to this region was found in the authors’ efforts to replicate this finding in a meta-analysis consisting of Old Order Amish, African American, and Caucasian subjects [23]. However, in a later effort to fine-map the MYP14 locus in a combined cohort of Old Order Amish and Ashkenazi Jewish families, replication of this locus was accomplished, and the QTL was narrowed to a 10 Mb area extending from chromosome 1p34.2 to chromosome 1p35.2 [24].

As was the case with localizing MYP14, successful efforts to find QTLs for refractive error have often used homogenous populations in genome-wide linkage studies. These homogenous populations have included Ashkenazi Jews [22–25], Caucasians [23,26], African Americans [23,26], and Old Order Amish [23,27], and different loci have been identified. For instance, MYP14 was not found in Caucasians, and 4q21 and 12q24 were found in Caucasians but not Ashkenazi Jews and Old Order Amish [21,22,28]. Overall, the QTL studies to date used either a small number of multigenerational pedigrees or twins. A more comprehensive and large-scale approach will help verify the existing regions.

This study is an international collaborative effort combining high-grade myopia pedigrees from five sites, leading to the largest family data set for myopia to date. We performed a genome-wide quantitative trait linkage scan using SPH and SEM directly and compared the results to known myopia loci. The outcomes of this study provide us with additional information regarding known genetic loci, and we have identified new myopia loci.

## Methods

### Patients and families

As previously described [29], 254 multiplex families (at least two affected individuals per family) consisting of 1,411 subjects (47% male) were ascertained independently at five international sites. Collaboration in this retrospective analysis occurred after each program had already recruited subjects. For the quantitative analysis presented here, we analyzed the largest subset of this data, which comprised 225 Caucasian families and 1,168 subjects (47% male). Participating centers included Cardiff University in the UK (CARD), Duke University Medical Center in the United States (DUK), the Kennedy Institute of National Eye Clinic at Hellerup, Denmark (HEL), the University of Melbourne in Australia (MEL), and Toulouse University in France (TOU). Before recruiting subjects, all study sites obtained the appropriate institutional review board human subjects research study approvals. All principles of the Declaration of Helsinki were adhered to. Individuals were not included in the study if they had any ocular disease or insult that could predispose to myopia, such as retinopathy of prematurity, retinal dystrophy, corneal keratopathy, and any genetic syndromes that include myopia as a clinical phenotypic component. Licensed ophthalmologists or optometrists conducted complete eye examinations on all consenting subjects. At each study site, subjects filled out clinical and family history questionnaires. SPH and SEM quantitative phenotypes were obtained for each individual.

### Sample preparation and genotyping

All subject genomic DNA samples were cataloged at the Duke University Center for Human Genetics (Duke CHG) and genotyped at the National Institutes of Health Center for Inherited Disease Research (CIDR). Most samples were extracted from blood (77.36%), while the remaining samples were derived from buccal mucosa (22.16%) or saliva specimens (0.48%). The genotyping platform used in this study was the Illumina Linkage Panel IVb consisting of 6,008 genome-wide single nucleotide polymorphisms (SNPs; Illumina). Following the CIDR genotyping protocol, each 96-well DNA sample plate included two Centre d’Etudes du Polymorphisme Humain (CEPH) DNA control samples and four replicates of subject DNA samples. In all, 1,411 subject DNA samples, 81 anonymous DNA replicates, and 87 CEPH DNA controls were genotyped in this study. The genotype data were transferred to the Duke CHG for data analysis.

### Quality control and data cleaning

Following CIDR genotyping protocol, several quality control measures were implemented to determine the final set of markers released to the Duke CHG for analysis [29]. A total of 5,928 SNPs were released by the CIDR for data analysis. Of these markers, 5,903 had GeneCall scores (a measure of how close a genotype is to the center of the cluster of other samples assigned to the same genotypes) greater than 0.15, and the 5,903 markers were taken forward for analysis in the pedigrees.

To examine family relationships using RELPAIR [30,31] and PREST [32], 700 markers with approximately equal inter-marker distances across the genome were selected. All family relationship errors were subsequently corrected, and the PEDCHECK software program [33] was used to again check for Mendelian inconsistencies. When we found inconsistencies, we followed one of two options: 1) we assigned the missing genotypes within a family for the members directly involved in the Mendelian inconsistencies, or 2) we dropped an individual from further analysis if the family designations were ambiguous and therefore could not be reconciled.

All SNPs were tested for the Hardy–Weinberg equilibrium (HWE). Two data sets with unrelated samples were formed in which one affected individual sample per family was randomly selected to cluster within a designated affected group and one unaffected individual sample per family was selected to add to a designated unaffected group. The HWE was then assessed using an exact test implemented in the Genetic Data Analysis (GDA) program [34]. Using 3,200 permutations (the default setting for the GDA program), we estimated empirical p values for each marker. We used the Q-VALUE program to correct for multiple testing [35]. A marker with a q value less than 0.2 was declared a significant deviation from the HWE and was excluded in the linkage analysis. At this point, 5,744 markers remained. To account for linkage disequilibrium among SNPs, we used LDSelect to determine tagging SNPs for analysis with an r^2^ cutoff of 0.16 [36]. Subsequently, we identified 4,656 tagging SNPs that comprised the final marker set that we defined for linkage analysis.

### Whole genome quantitative trait linkage analysis

For this analysis, we investigated two phenotypes (SPH and SE) that were computed as the binocular average of measurements of the right and left eyes. These binocular averages for SPH and SEM were used directly for the quantitative analysis. When individuals have great discrepancies in SPH or SEM measurements between the eyes, this averaging can result in a muted linkage signal. For our data set, however, most individuals had similar SPH and SEM values for both eyes. Which refractive error parameter is the “correct” one to use for refractive error genetics study is unknown; therefore, we chose to use both measurements in our analyses. SPH and SEM are surrogates for axial length—a metric that was not consistently obtained for participants. Of the 1,004 individuals with SPH measurements available for both eyes, only 37, or 3.7%, could be classified as having one normal eye and one moderately myopic eye (−1.00 OD ≤ SPH≤−3.00 OD), and only two, or 0.2%, could be classified as having one normal or moderately myopic eye and one highly myopic eye (SPH≤−6.00 OD). For SE, the percentages of different myopia classifications between the two eyes were 2.1% and 0.3%, respectively. Individuals who were missing a measurement in at least one eye were assigned as missing for the phenotype of interest. We used the Sequential Oligogenic Linkage Analysis Routines (SOLAR) variance components procedure to perform quantitative trait linkage analysis using 4,656 tagging SNPs across the genome [37]. Under this procedure, the quantitative phenotypes SPH and SEM were defined as linear functions of the *n* QTLs (*r_i_*) that influence the trait:

$$SPH\;\left(SE\right)=µ\;+\;X\beta \;+\;\sum \;r_{i}\;+\;e$$

where **X** is a matrix of covariates and β is the regression coefficient matrix for the covariates. Parameters that comprise the likelihood function of SPH or SEM include the identical by descent (IBD) probability for a marker linked to a QTL, the additive genetic variance attributable to the unobserved QTL (σ^2^*_q_*), and other variance components. Using a likelihood-ratio test, SOLAR tests a null hypothesis of no linkage (σ^2^*_q_*=0). SOLAR also calculates a logarithm of the odds (LOD) score as evidence of linkage to an individual marker for two-point analysis or to an imputed chromosomal position for multipoint analysis.

Before calculating the likelihood, locus-specific IBD information was computed for all pairs of relatives [37]. Following the premise of the Fulker et al. [38] method, the SOLAR multipoint mapping strategy uses the map distance between markers to compute the IBD information for a pair of relatives at a QTL linked to a marker. For our analysis, we used a Kosambi sex-averaged map obtained from deCode [39]. The factor representing ascertainment sites was included as a house effect in our model to adjust for ascertainment bias. That is, potential differences across ascertainment centers were investigated in our analysis by treating center as a random effect in the variance component model. We originally included sex as a covariate in the polygenic model, but dropped it from the final model due to a lack of significance. We examined the normality of phenotypic distribution before the linkage analysis using a Q-Q plot to meet the underlying assumption of the variance component model. We investigated several transformations, and viewed plots of log-transformed data in Q-Q plots. To meet the criterion of normality, we used the following formulas: SPH_new_=4.3xlog_10_[-(SPH-0.25)] and SE_new_=4.4xlog_10_[-(SE-0.25)].

## Results

Of 254 families typed by the CIDR, the largest subset comprised 225 Caucasian families. This subset included 1,168 subjects. SPH data for only one eye were available for 164 subjects, and 256 subjects had SEM data for only one eye. These individuals were therefore not included in quantitative phenotype SPH or SEM analytical assessments. Summary descriptive statistics information for the samples included in the QTL analysis is provided in Table 1.

**Table 1:** Data set Descriptive Statistics by Mean Refractive Error.

**Characteristic**	**Mean sphere data set**	**Mean spherical equivalent data set**
Total Sample Size	1004	912
Number of Families	216	215
Average Family Size ± SD	4.65±3.20	4.24 ±3.06
Average Age of Subject ± SD (years)	42.55±19.97	43.10±20.08
Number with Age Data	923	838
Mean Refractive Error ± SD (Diopters)	−5.74±5.66	−5.36±5.57
Heritability (h^2^)	0.232±0.0720	0.339±0.0772

The heritability of SPH or SEM was highly significant (p=0.0001298 and p=0.0000006 for SPH and SE, respectively), with heritabilities of 23.2% (SPH) and 33.9% (SE). We used a threshold level of LOD≥1.50 to highlight promising initial linkage regions of interest for two-point and multipoint linkage analyses (Table 2 and Table 3) [40].

**Table 2:** Summary of peak regions with LOD > 1.5 from two-point QTL analysis.

**Chromosome**	**Marker**	**DECODE (cM)**	**Mean sphere**	**Mean spherical equivalent**
1	rs720887	103.33	1.09	1.68
2	rs925229	47.88	0.97	1.62
2	rs1369842	198.07	1.42	1.71
3	rs1500530	128.84	0.72	1.6
5	rs925893	129.69	0.33	1.54
5	rs4868073	183.33	1.27	1.52
	rs472959	187.27	1.11	1.62
6	rs4960147	15.18	1.56	1.36
6	rs2213661	55.45	1	1.65
6	rs2000203	87.57	1.76	1.19
	rs3798425	88.55	0.84	1.86
	rs1457947	89.14	1.52	1.24
	rs932492	90.62	1.22	1.88
	rs1059306	93.3	1.3	1.76
	rs1179900	96.1	0.6	2.13
	rs491112	100.5	0.35	1.65
7	rs28156	96.18	1.33	2.09
11	rs731365	40.36	1.02	1.96
11	rs528638	132.61	1.13	1.81
	rs570969	150.15	0.72	1.54
12	rs417664	15.85	0.91	1.54
12	rs2730550	138	0.9	1.84
15	rs278357	22.65	1.17	1.73
15	rs1445020	71.05	0.28	1.58
15	rs7168948	133.2	1.55	1.59
18	rs770238	6.63	1.04	1.6
19	rs1715093	12.18	1.13	1.67
19	rs1122713	36.79	1.38	1.95
22	rs2399153	1.54	1.19	1.53

CentiMorgans=cM, DECODE mapping position used.

**Table 3:** Summary of the peak Regions with LOD > 1.5 from multipoint quantitative trait Locus analysis.

**Chromosome**	**Locus**	**Marker**	**DECODE (cM)**	**Mean spherical equivalent**
1	1p31.1	rs655938	98	
	(near MYP14, 1p36)	Peak LOD score	100	1.69
		rs1389790	103	
2	2q33.1	rs997467	197	
	(near MYP12, q37.1)	Peak	198	1.25
		rs970595	199	
3	3q29	rs790927	214	
		rs1864668 (Peak)	221	1.07
5	5p13.3	rs2034586 Peak		
	(near MYP16,5p15.33–15.2)	rs1021711	49	1.51
5	5q35.1–35.2	rs1054998	180	
		Peak	188	2.05
		rs1875189	192	
6	6q13	rs1817255	85	
		Peak	88	1.96
	6q16.1	Peak	95	1.57
		rs1040155	100	
7	7q11.23	rs3135677	87	
	to	Peak	97	1.19
	7q21.2	rs9008	102	
10	10p11.21	rs913167	63	
	To	Peak	64	1.68
	10q11.22	rs733488	66	
11	11q24.2	rs1944819	137	
		Peak	142	0.54
		rs570969	148	
12	12q24.31	rs922873	145	
	(near MYP3, 12q21–23)	Peak	151	0.63
		rs1388149	152	

CentiMorgans=cM.

### Quantitative trait loci linkage regions for the sphere phenotype

**Figure 1:**
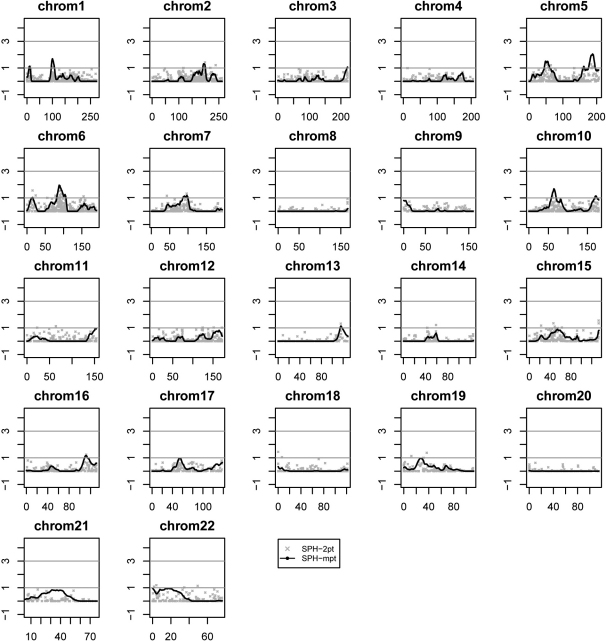
Genome-wide linkage analysis results for mean sphere (SPH) measurements. Logarithm of the odds (LOD) scores are plotted on the y-axes and genetic distance in centiMorgans (cM) along each chromosome on the x-axes. Two analyses are shown: two-point and multipoint quantitative trait linkage.

Four markers across two chromosomes resulted in two-point LOD scores ≥ 1.5 (Table 2). Graphical results for the genome-wide linkage scan are depicted in Figure 1, and promising linked markers and linkage regions are summarized in Table 2 and Table 3. The rs2000203 at 87.57 centiMorgans (cM; two-point LOD=1.76) and rs1457947 at 89.14 cM (two-point LOD=1.52) on chromosome 6 are in close proximity to each other. These markers also fall within a linkage region identified by the Caucasian subset in our data when treating SPH as a binary trait [29]. However, none of these four markers are near known MYP loci reported previously (Table 2).

**Figure 2:**
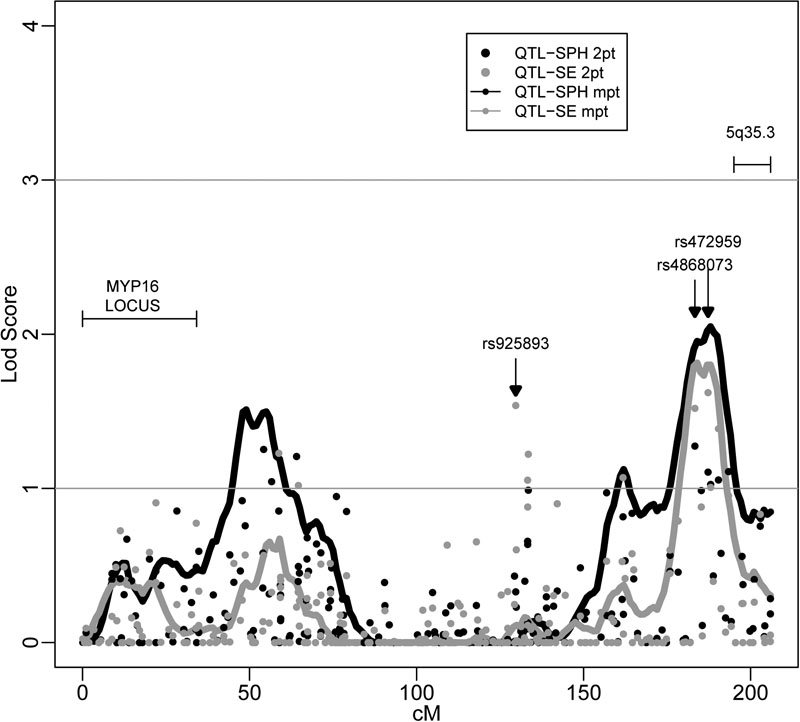
Chromosome 5 linkage analysis results for mean sphere (SPH) and mean spherical equivalence (SE). Multipoint (MPT) and two-point (2PT) logarithm of the odds (LOD) score results are presented.

Five linkage regions on separate chromosomes contained peak multipoint LOD scores ≥ 1.5 (Table 3). Figure 2 depicts a clear multipoint peak for chromosome 5q35.1–5q35.2 extending from 180 to 192 cM with a maximum LOD of 2.05, which is supported by rs4868073 (183.33 cM) with a two-point LOD score of 1.27. The second highest multipoint peak is on chromosome 6q13–6q16.1 (peak LOD=1.96, 88 cM), which showed a LOD score over 2 when the SEM was analyzed (see next section). Other regions of interest include chromosome 1p31.1 (peak multipoint LOD=1.69 at 100 cM), chromosome 10p11.21–10q11.22 (peak multipoint LOD=1.68 at 64 cM), and chromosome 5p13.3 (peak multipoint LOD=1.51 at 49 cM; Figure 2). The chromosome 1p31.1 locus is close to the MYP14, located at 1p36, and the 5p13.3 locus is near a known myopia locus, MYP16, located at 5p15.33–5p15.2.

### Quantitative trait loci linkage regions for the spherical equivalent phenotype

**Figure 3:**
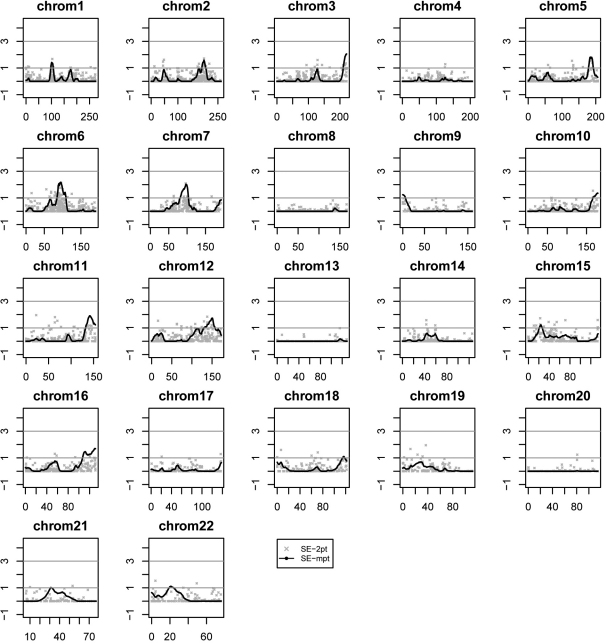
Genome-wide linkage analysis results for mean spherical equivalent (SE) measurements. Logarithm of the odds (LOD) scores are plotted on the y-axes and genetic distance in centiMorgans (cM) along each chromosome on the x-axes. Two analyses are shown: two-point and multipoint quantitative trait linkage.

**Figure 4:**
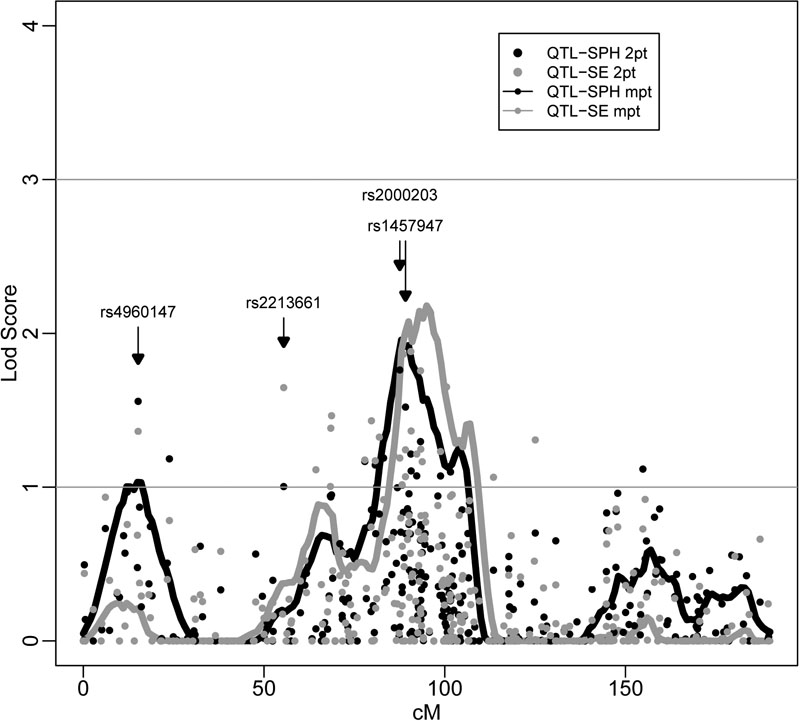
Chromosome 6 linkage analysis results for mean sphere (SPH) and mean spherical equivalence (SE). Multipoint (MPT) and two-point (2PT) logarithm of the odds (LOD) score results are presented.

The linkage analyses of the SEM derived more markers and linkage regions of interest. There are 26 markers across 12 chromosomes that resulted in two-point LOD scores ≥1.5, and eight linkage regions from eight chromosomes that contained peak multipoint LOD scores ≥1.5 (Table 2 and Table 3, and Figure 3). Similar to the SPH phenotype, the strongest evidence of linkage for the SEM occurred at chromosome 6q13–6q16.1 (peak two-point LOD=2.13 at rs1179900 and peak multipoint LOD=2.18 at 95 cM) and chromosome 5q35.1–5q35.2 (peak two-point LOD=1.62 at 187.27 cM and peak multipoint LOD=1.80 at 188 cM). In particular, the chromosome 6q13–16.1 region is strongly supported by a set of seven markers with two-point LOD scores ranging from 1.19 to 2.13 (Table 2). The overall view of the two-point and multipoint linkage results for chromosome 6 is depicted in Figure 4.

Two more regions showed LOD scores above 2: chromosome 3q29 (multipoint LOD=2.05 at 221 cM) and chromosome 7q11.23–7q21.2 (multipoint LOD=2.03 at 97 cM; Table 3). Overall, six out of 11 regions showed similar LOD score trends for the SPH and the SEM.

Among the 11 linkage regions outlined in Table 3, four loci are in close proximity to previously identified myopia loci. They are chromosome 1p31.1 for MYP14, chromosome 2q33.1 for MYP12, chromosome 5p13.3 for MYP16, and chromosome 12q24.31 for MYP3.

## Discussion

We report a large-scale refractive error quantitative trait linkage study that used dense SNP genotyping as opposed to microsatellite markers. In this study, we identified two loci of high interest linked to myopic refractive error: (1) chromosome 6q13–6q16.1 with multipoint peak LOD scores of 1.96 for SPH and 2.18 for SEM and (2) chromosome 5q35.1–5q35.2 with multipoint peak LOD scores of 2.05 for SPH and 1.80 for SEM. The consistency of the LOD score significance derived from two different but similar measures of refractive error, SPH and SE, for these two loci. The chromosome 5q35.1–35.2 locus overlaps with a region that we reported previously using high myopia as a qualitative disease phenotype [29]. The chromosome 6q13–6q16.1 locus has not been previously reported as a linkage region for myopia in the literature.

Overall, our LOD scores are low (not achieving the standard significant threshold LOD ≥3) [41]. This is expected as we determined a relatively low residual heritability (24%–25%) of refractive error for our data set. Furthermore, since quantitative traits likely result from a confluence of factors including the involvement of several polymorphic genes and environmental conditions, the signal at a single QTL will not appear as strong given other factors acting on the trait. Despite these limitations, the pattern of our results still provides a good overview of refractive error loci across the genome.

In conducting our analysis, we recognized that an investigation of axial length, a major determinant of axial myopia, in our data might be insightful. Unfortunately, axial length data were collected for fewer than a third of our study participants (total sample size=266 compared to 1,004 and 912 for SPH and SE, respectively). Quantitative trait linkage analysis was conducted for the axial length data set, but no two-point or multipoint results exceeded a LOD threshold of 1.5. The power to detect a linkage signal for axial length was severely hampered by the low sample size.

In our previous report of linkage regions for high-grade myopia, the linkage regions were largely inconsistent between the analyses of the disease states defined by the SPH and the SEM [29]. When SPH and SEM are analyzed as quantitative traits, consistent results were found. This is expected, as the spreads of the SPH and SEM distributions are similar (SD: ±5.66 [SPH] versus ±5.57 [SE]). Most importantly, this shows the advantage of using quantitative traits in analyses. Comparatively, dichotomizing a distribution to a binary variable tends to lose power and adds phenotype state uncertainty when different clinical definitions of the affection status exist (e.g., SPH versus SE).

The present study provides important information. First, two new loci on chromosomes 5 and 6 are likely to be new chromosomal regions that link to myopic refractive errors. Second, our linkage analysis again replicated suggestive significance of the MYP3 locus on chromosome 12q. Clearly, this locus should undergo additional scrutiny in other data sets. Third, our analysis underscores the benefits of using refractive error as a quantitative trait for linkage, and demonstrates the advantage of using an SNP-based linkage screening protocol with higher marker density than conventional microsatellite markers to map new loci. The results of the present study of a large family data set using high-density SNPs for linkage scanning should aid in triaging candidate genes and loci for future genome-wide association studies and deep sequencing efforts.
